# Evaluation of the real-time fluorescence loop-mediated isothermal amplification assay for the detection of *Streptococcus agalactiae*

**DOI:** 10.1042/BSR20190383

**Published:** 2019-05-14

**Authors:** Xu-Guang Guo, Ya-Ru Zhuang, Jin-Zhou Wen, Tian-Ao Xie, Ye-Ling Liu, Guo-Dong Zhu, Yong Xia

**Affiliations:** 1 Department of Clinical Laboratory Medicine, the Third Affiliated Hospital of Guangzhou Medical University, Guangzhou, Guangdong, China; 2 Key Laboratory for Major Obstetric Diseases of Guangdong Province, the Third Affiliated Hospital of Guangzhou Medical University, Guangzhou, Guangdong, China; 3 Center for Disease Control and Prevention of Chaozhou, Chaozhou, Guangdong, China; 4 Department of Clinical Medicine, the Third Clinical School of Guangzhou Medical University, Guangzhou, China; 5 Department of Clinical Laboratory Medicine, The Third Affiliated Hospital of Guangzhou Medical University, Guangzhou, Guangdong, China

**Keywords:** LAMP, loop-mediated isothermal amplification, real-time fluorescence loop-mediated isothermal amplification, real-time fluorescence quantitive PCR, Streptococcus agalactiae

## Abstract

*Streptococcus agalactiae* is a major pathogenic bacterium causing perinatal infections in humans. In the present study, a novel real-time fluorescence loop-mediated isothermal amplification technology was successfully developed and evaluated for the detection of *S. agalactiae* in a single reaction. Six specific primers were designed to amplify the corresponding six regions of fbs B gene of *S. agalactiae*, using Bst DNA polymerase with DNA strand displacement activity at a constant temperature for 60 min. The presence of *S. agalactiae* was indicated by the fluorescence in real-time. Amplification of the targeted gene fragment was optimized with the primer 1 in the current setup. Positive result was only obtained for Sa by Real-LAMP among 10 tested relevant bacterial strains, with the detection sensitivity of 300 pg/µl. Real-LAMP was demonstrated to be a simple and rapid detection tool for *S. agalactiae* with high specificity and stability, which ensures its wide application and broad prospective utilization in clinical practice for the rapid detection of *S. agalactiae*.

## Introduction

*Streptococcus agalactiae* (Sa), a facultative anaerobic Gram-positive *Streptococcus*, was reported to colonize the human gastrointestinal and genitourinary tract of up to 30% of healthy human adults. Moreover, Sa was regarded as a major pathogenic bacterial strain for perinatal infections in pregnant women and their newborns as early as in 1970s. It was the leading cause for sepsis and meningitis in infants and young children, and has been a serious concern in perinatal medicine. Therefore, a rapid, specific, economical and reliable detection technique is particularly required to facilitate the detection of Sa in clinical samples [1].

A recently developed nucleic acid amplification technology, loop-mediated isothermal amplification (LAMP), has been utilized for the detection of various pathogens in seafood and clinical samples, which was first proposed by Notomi et al. in [2] with the amplification of 109-fold from single digital copies of the targeted gene. The technology was a PCR-based reaction conducted at a constant temperature (60–65°C) for 30–60 min using a DNA polymerase with strand displacement activity (Bst DNA polymerase). The reaction rate was further increased two to three times by Nagamine et al. [3], and two loop primers were specially designed and adopted. This significantly increased the detection sensitivity as compared with conventional PCR, and the reaction time was greatly reduced. Till date, LAMP technology has been successfully applied for the detection of multiple bacterial and viral strains, demonstrating its significant application potential. A novel real-time LAMP (Real Time-LAMP or Real-LAMP) was developed, and first utilized for the diagnostic detection of malaria by Naomi et al. in 2011, with the detection sensitivity of 96.7% and specificity of 91.7%. Meanwhile, this technology was also tested for HBV detection in China.

Though LAMP technology has been utilized for Sa detection in aquaculture research, including samples from the red tailed crown fish and tilapia in China [4], Sa detection based on Real-LAMP has not yet been reported. Therefore, in this study, a Real-LAMP-based approach was established for Sa detection, which combined the high sensitivity of real-time fluorescent detection and the short detection time course of LAMP, to investigate its feasibility in clinical practice and facilitate the rapid detection of Sa in clinical samples.

## Methods

### Bacterial strains and bacterial genomic DNA extraction

The strains were obtained from the Department of microbiology laboratory, the Third Affiliated Hospital of Guangzhou Medical University, where the strains were isolated, identified by VITEK 2 automatic microorganism identification instrument, and stored at −70°C. The strains used in the present study included *Acinetobacter baumannii coli, Pseudomonas aeruginosa, Cryptococcus rolland, Streptococcus agalactiae, Staphylococcus aureus, Escherichia coli, Staphylococcus epidermidis, Stenotrophomonas maltophilia, Klebsiella pneumoniae, Streptococcus pyogenes* and *Vibrio parahemolyticus*. Bacterial strains stored at −70°C were inoculated onto blood agar plates and incubated at 37°C for 18–24 h. The bacterial colonies on the agar plates were picked and inoculated into sterile normal saline to prepare 1 ml bacterial suspensions. The genomic DNA was extracted according to the instruction manuals of the kits.

### Primer design and Real-LAMP system

The primers were designed using the LAMP primer design software Primer Explorer V4 according to the DNA sequence of fbs B gene of Sa (Tables 1 and 2). The primers fbs B-1 and fbs B-2 were synthesized by Invitrogen (Shanghai, China). LAMP reaction system was established according to the manual of DNA amplification. One drop of wax oil was added after the reaction volume was set up, before adding the fluorescent dye. The working solution consisted of FIP, BIP, F3, B3, LF and LB in the ratio of 8:8:1:1:4:4. The final concentration of each primer was 1.6 μmol/l, 1.6 μmol/l, 0.2 μmol/l, 0.2 μmol/l, 0.8 μmol/l and 0.8 μmol/l, respectively, in the total reaction volume of 25 µl. The reaction was conducted in ABI7500 PCR machine at 63°C for 60 min. Sterile distilled water was used as negative control in all the tests. The fluorescence channel was selected for SYBR detection. The LAMP working solution was prepared by mixing the internal, external and cyclic primers in the ratio of 8:1:4.

**Table 1:** DNA oligonucleotide primer 1 sequence of LAMP for the fbs B gene

Name	Primer (5′-35′)	Primer length (bp)
Upstream (FIP)	CAACTGGTTGAACCGGAGCTCTCCACAACAAAGTTCAACTG	41
Downstream (BIP)	GCAGCACATGATGCCATTTCAGAGGTCATTTCCGCAGTTG	40
Upstream (F3)	AGAAATCTGAAACTGTGGCA	20
Downstream (B3)	TCCCTAAAGCTTTCTCAACATC	22
Upstream (LF)	CTGTTGGACAGAAGTTTGCG	20
Downstream (LB)	TTGCTAATACAGCCGGTGTAA	21

**Table 2:** DNA oligonucleotide primer 2 sequence of Real-LAMP for the fbs B gene

Name	Primer (55′-35′)	Primer length (bp)
Upstream (FIP)	CAACTGGTTGAACCGGAGCTCTCCACAACAAAGTTCAACTG	41
Downstream (BIP)	CAGCACATGATGCCATTTCAGCAGGTCATTTCCGCAGTTG	40
Upstream (F3)	AGAAATCTGAAACTGTGGCA	20
Downstream (B3)	TCCCTAAAGCTTTCTCAACATC	21
Upstream (LF)	CTGTTGGACAGAAGTTTGCG	20
Downstream (LB)	TTGCTAATACAGCCGGTGTAA	21

### Primer screening experiment

Two sets of primers, fbs B-1 and fbs B-2, were prepared as working solutions. The amplification efficiency between the two primers was compared using Sa DNA as the template. The sampling as well as the experimental setups was in accordance with the instructions of the DNA amplification kit. The primer with higher amplification efficiency was used in the subsequent assays. The working solutions were prepared using the selected primers. Two separate Real-LAMP reactions were set up using working solution with or without ring primer, to amplify fbs B gene of Sa and compare the amplification curves.

### Specificity test of LAMP

The genomic DNA of Sa and other common pathogenic bacteria (including *Acinetobacter baumannii coli, Pseudomonas aeruginosa, Cryptococcus rolland, Staphylococcus aureus, Escherichia coli, Staphylococcus epidermidis, Stenotrophomonas maltophilia, Klebsiella pneumoniae, Streptococcus pyogenes* and *Vibrio parahaemolyticus*) were extracted and amplified according to the Real-LAMP conditions. The detection signals and the specificity of primers were evaluated.

### Sensitivity test of LAMP

The concentration of Sa genomic DNA was measured using the Thermo Scientific Nanodrop 2000 spectrophotometer after titering with 1 µl of TE buffer. The sensitivity of the reaction was assessed using a serial dilution of the genomic DNA template, wherein the original 10 µl of genomic DNA was sequentially 10-fold diluted three times. The Real-LAMP reactions with identical total volume and the different DNA templates with four concentrations were performed under identical conditions, and the sensitivity of detection was evaluated according to the amplification curves. Meanwhile, the quality of primers was tested by examining the formation of primer dimers based on the dissolution curves. The inference of primer dimer formation was excluded in the present study.

### Repeatability test of LAMP

The genomic DNA from one positive bacterial strain and one negative bacterial strain was used for repeatability assessment of Real-LAMP reaction. The experiments were repeated three times under identical conditions.

## Results

### Primer screening experiment

**Figure 1:**
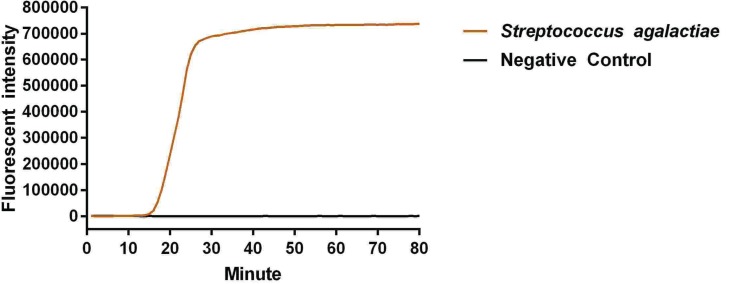
Primer screening experiment using primer 1

**Figure 2:**
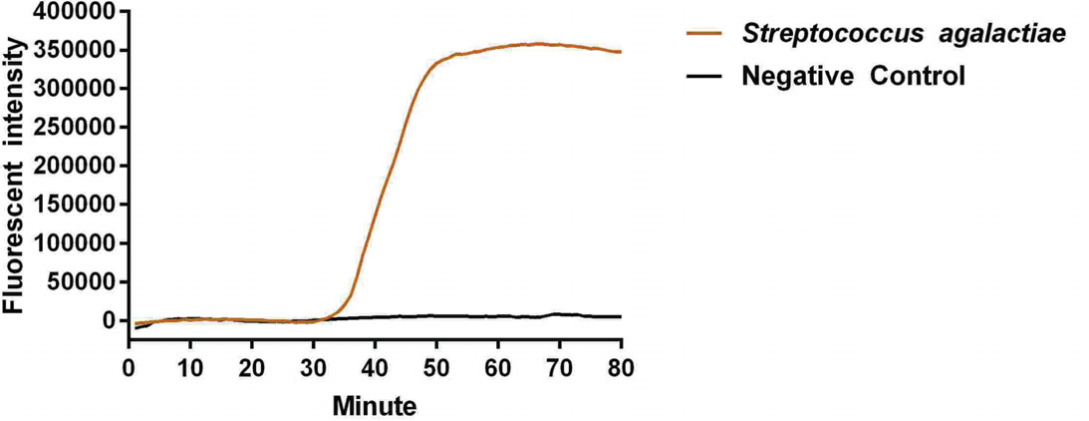
Primer screening experiment using primer 2

The reaction peak of fbs B gene amplification was visually detected by ABI 7500 at 17 and 35 min after reaction initiation, using primers fbs B-1 and fbs B-2, respectively (Figures 1 and 2). The visually detectable reaction peak emerged earlier in the reaction with primer fbs B-1 as compared with primer fbs B-2; and the fluorescent intensity was also higher in the reaction with primer fbs B-1 than with primer fbs B-2 at each check-point. In addition, no false positive result was found in the negative control. Therefore, primer fbs B-1 had relatively higher amplification efficiency for Sa fbs B gene and was selected for the subsequent experiment. In addition, no reaction peak in the Real-LAMP without ring primer could be visually detected until 63 min after initiation.

### Specificity test of LAMP

**Figure 3:**
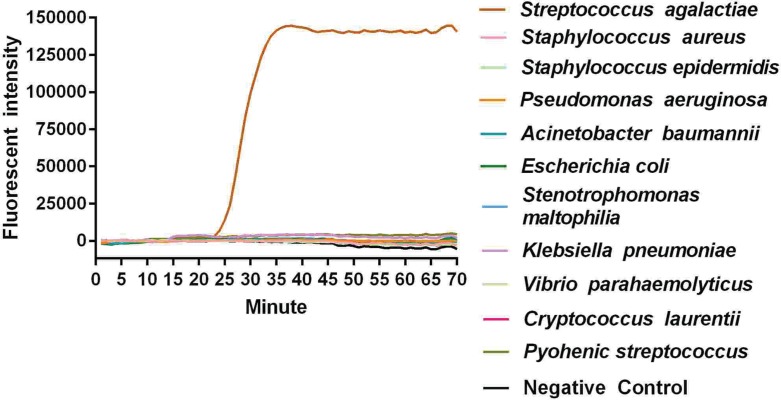
The specificity experiment of Real-LAMP

There was no specific amplification using templates from other common pathogenic bacteria, indicating satisfactory specificity for Sa (Figure 3). Therefore, the primers for Real-LAMP in the present study had satisfactory specificity, without cross-reactivity with other non-targeted bacteria.

### Sensitivity test of LAMP

**Figure 4:**
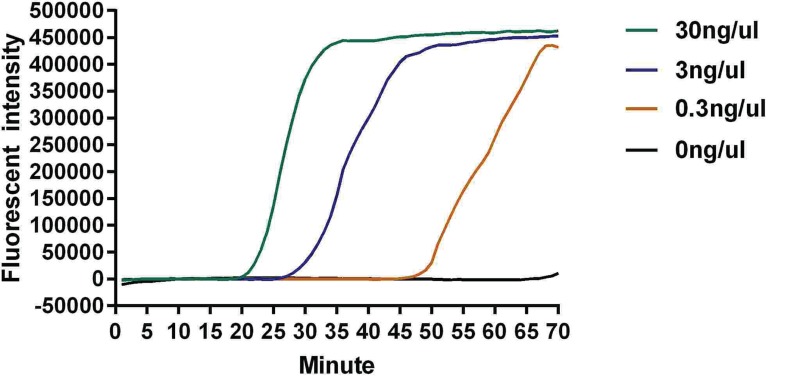
The sensitivity experiment of Real-LAMP

The concentration of Sa genomic DNA was 30 ng/µl, which was serially diluted three times by 10-fold to 3 ng/µl, 300 pg/µl and 30 pg/µl. As shown in Figure 3, fbs B gene was visually detected at 22, 42 and 52 min after reaction initiation with DNA template at 30 ng/µl, 3 ng/µl and 300 pg/µl, respectively. The sensitivity of the detection was 300 pg/µl (Figure 4). No double-peak appeared on the melting curve with the DNA template at the three concentrations, indicating the absence of dimer formation of primers and that the result was reliable.

### Repeatability test of LAMP

**Figure 5:**
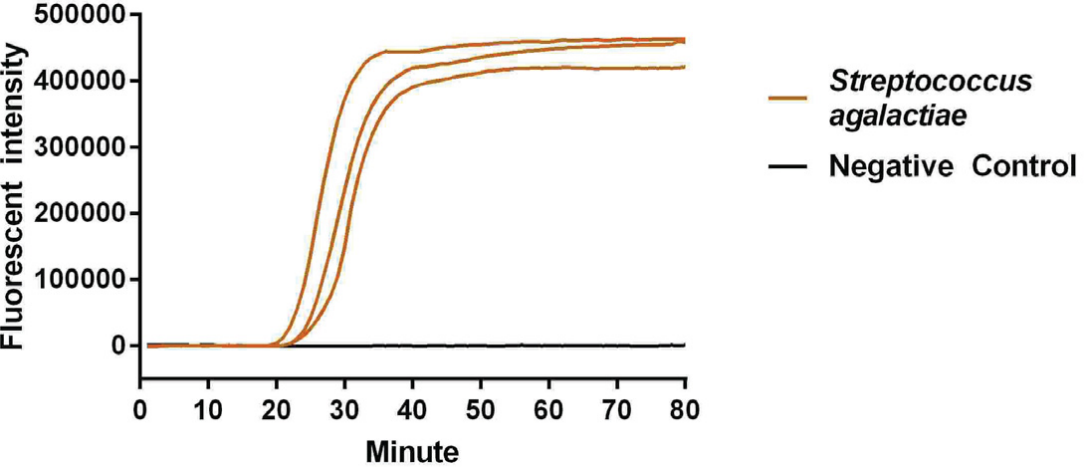
The repeatability experiment of Real-LAMP

The reaction peak was visually detected almost simultaneously for the triplicate tubes of positive sample, and the amplification curves nearly overlapped (Figure 5). Meanwhile, the CT value for positive control was 23.15, 23.42 and 23.24, respectively, for triplicate tubes, with the CV of 0.59% (<5%), suggesting satisfactory repeatability of Real-LAMP.

## Discussion

Sa is the most common genital tract pathogen, the leading cause for neonatal pneumonia deaths, perinatal infection of pregnant women and their newborns, sepsis and meningitis of infants and young children [5]. However, rapid and reliable detection of Sa infection is an unmet medical need in clinical practice. Therefore, a rapid, specific, sensitive and cost-effective diagnostic methodology was developed in the present study based on Real-time LAMP technology.

Real-time LAMP is the combination of real-time fluorescent technology and isothermal amplification technology, with the advantages of being simple, rapid and highly specific. The whole process of amplification could be dynamically monitored through Real-LAMP. The high specificity and enhanced amplification efficiency by adoption of multiple specific primers was the unique advantage of LAMP.

The high specificity of Real-LAMP relied on well-designed primers, which ensured strict alignment between primers and targeted region, not only facilitating major amplification of the targeted sequence but also differentiating one-base pair-difference among all similar templates; to avoid any interference with non-targeted DNA sequences [6]. In the present study, LAMP was performed to detect the presence of DNA samples for Sa and 10 other strains of common pathogenic bacteria. Positive amplification was only observed in Sa, but not in the other 10 strains, indicating high specificity of LAMP primers and no cross-reactivity with other non-targeted strains. In addition, the ring primers against the target sequence could increase the reaction rate and significantly reduce the reaction time. The reaction rate was increased by 3-fold; the positive readout could be visually detected at 17 min after reaction initiation, and the whole reaction could be completed within 45 min, demonstrating significantly reduced detection time as compared with conventional PCR.

The readout of conventional LAMP was the white precipitates and dependent on visual inspection. However, weak positive results obtained due to low amplification efficiency or minute amount of templates were usually very challenging for visual inspection [7–9]. To overcome this challenge, the fluorescent dye SYTO-9 was used in the present study and the ABI7500 system was employed. The real-time fluorescent quantitative PCR instrument was utilized as LAMP platform, which allowed dynamic observation with increased detection sensitivity. However, the SYTO-9 dye could bind to double-stranded DNA if there was dimer formation of primers, which could lead to false-positive result. To test for potential false-positive results, the melting curve was analyzed and no double-peak was found, indicating no primer dimer formation during the reaction and high specificity of our assay setup.

Though Real-LAMP is a highly sensitive detection technology, it has some limitations. For instance, aerosol pollution was the main cause of false-positive in Real-LAMP [10,11]; the influence of aerosol diffused into the reaction system was sometimes significant due to the high sensitivity of this technique. The fluorescent dye was usually added into the reaction when LAMP was completed, which might result in false-positive because of lid-opening related LAMP product diffusion. In the present study, the SYT0-9 fluorescent dye was added before the reaction to avoid the lid opening step, which can induce aerosol pollution. The lid was opened once and the whole process was completed with the closed lid. Therefore, aerosol pollution from amplification products was effectively prevented. In order to avoid false-positive results, preventive measures should be considered such as ventilation during operation, avoiding repeated lid opening and processing reaction product timely. In addition, the pipette tips should be frequently changed during the reaction system set up.

Molecular biology-based techniques, such as PCR and real-time PCR assays, have been successfully established for detection of Sa. However, the PCR-based techniques relied on sophisticated apparatus including high precision temperature cycler and gel imager or complex sample-handling procedures, which limited their application in primary medical facilities or epidemic outbreaks requiring rapid ‘on-site’ detection screening. Although temperature cycler is not required for self-sustained sequence replication and nucleic acid sequence-based amplification, the low amplification temperature and poor specificity make it difficult for clinical application. Strand displacement amplification has overcome all the drawbacks of the above assays, but the non-specific nucleic acid sequences within the samples may lead to non-specific amplification. Even though the irrelevant DNA templates could be removed by treating with restriction enzymes, strong background signal was induced simultaneously, which was very difficult to remove afterward [12]. Given the drawbacks and limitations of conventional nucleic acid amplification technologies, the establishment of alternative methodologies for improved sensitive and specific detection of Sa was needed, and LAMP was an optional approach.

There are multiple merits of LAMP technology for detecting pathogenic bacteria. First, LAMP has high specificity with no interference by non-targeted DNA sequences; and high efficient amplification occurred once the special regions were strictly recognized by six well-designed primers [13,14]. Second, trace amount of template as low as fg/ml could be detected by LAMP, the sensitivity of LAMP was approximately 1–2 orders of magnitude folds as compared with other technologies. However, it was also reported that the sensitivity was not significantly increased by LAMP, which might be associated with the differences in manipulation processes adopted by different research groups. Third, the reaction rate was increased by LAMP; and few copies of the targeted gene could be amplified 109 times within 30–60 min. Constant temperature was utilized in LAMP without the time consuming steps of temperature alterations. In addition, ring primers were adopted in LAMP, which could significantly increase the reaction rate and further reduce the time and cost. Only one water bath or constant temperature metal bath was required for LAMP, and the readout could be directly visualized without any expensive instrument. Therefore, LAMP has broad applications in developing countries, and has been validated in Africa for HIV and malaria detection.

Some researchers have used sip gene instead of fbs-B and carried out the LAMP for the detection of *S. agalactiae*. Which gene is better in the detection of *S. agalactiae*, it needs further study [15].

As compared with conventional PCR, the working mechanism of LAMP was relatively complicated and the requirement for primer design and selection was very strict [16–20]. However, real-time LAMP offers dynamic monitoring and detection, and the samples with high concentration of targeted DNA could be detected within 30 min, which greatly reduced the turnaround time [21–25]. Overall, LAMP is a gene amplification technology that does not require special reagents or instruments, and has the advantages of speed, simplicity, high sensitivity and high specificity [26–30].

Given the limited number of samples in the present study, further validation is required with more samples. Meanwhile, since the DNA amount in the clinical samples was varied, and the DNA extraction process was easily interfered, the risk of obtaining non-specific DNA templates remained; so the assay will be repeated using clinical samples such as vaginal secretions for further validation. Optimization of DNA extraction from various clinical samples should be performed in order to extend the LAMP assay from laboratory testing to clinical detection.

In summary, Real-LAMP used in the present study for Sa detection was demonstrated to have advantages of sensitivity, specificity, speed and ease of operation, and could be considered as a novel detection alternative for Sa with the potential to be a routine clinical assay. The LAMP assay would be continuously improved with the advances in technology, and it is likely that it could be used in the clinical detection of Sa in the near future.

## Abbreviations

LAMP: loop-mediated isothermal amplification
Real-LAMP: real-time fluorescence loop-mediated isothermal amplification
Sa: *Streptococcus agalactiae*
